# Tail Docking of Piglets 1: Stress Response of Piglets to Tail Docking

**DOI:** 10.3390/ani10091701

**Published:** 2020-09-20

**Authors:** Rebecca Morrison, Paul Hemsworth

**Affiliations:** 1 Rivalea (Australia), Research and Innovation, Redlands Road, Corowa 2640, Australia; 2 Animal Welfare Science Centre, Faculty of Veterinary and Agricultural Sciences, University of Melbourne, Parkville, Victoria 3010, Australia; phh@unimelb.edu.au

**Keywords:** pigs, tail docking, pain, stress, welfare

## Abstract

**Simple Summary:**

Tail docking is a common industry practice in pork production to reduce tail biting in groups of pigs. Tail biting behaviour involves destructive chewing of the tails of group-mates, which can both compromise pig welfare and cause economic losses. The etiology of tail biting remains poorly understood and potential predisposing factors include crowding, poor ventilation, nutrition and health. This experiment examined the stress response of piglets to two common tail docking procedures: docking with either clippers or cauterisation, surgical castration and a sham handling treatment (handling alone). In comparison to the sham handling treatment, the two tail docking treatments increased the duration of vocalisations and number of escape attempts during treatment, the cortisol response at 15 min post-treatment and the duration of standing with head lowered in the first 60 min post-treatment. However, both these behavioural responses during treatment and the cortisol response at 15 min post-treatment to clippers or cauterisation were lower than that of castration. Piglets in the cauterisation treatment group also had a lower cortisol response at 30 min post-treatment than those in the clipper treatment group. The cortisol and behavioural responses in the tail docking and castration treatments had diminished by 23–24 h post-treatment.

**Abstract:**

This experiment compared the stress responses of piglets to tail docking. Two hundred and eighty-eight piglets were allocated to the following treatments at 2 d post-farrowing: (1) sham handling treatment; (2) surgical castration; (3) tail docking using clippers; (4) tail docking using a cauterising iron. Blood samples were collected at 15 min, 30 min and 24 h post-treatment and analysed for total plasma cortisol. Behaviours indicative of pain, such as escape attempts, vocalisations and standing with head lowered were measured. Cortisol concentrations at 15 min post-treatment were higher (*p* < 0.001) in the tail docking and castration treatment groups than the sham handling treatment group, but at 30 min post-treatment, only the clipper and castration treatment groups had higher (*p* < 0.001) cortisol concentrations than the sham handling treatment. Duration of vocalisations and escape attempts were greater (*p* < 0.0001) during the castration treatment than the sham and tail docking treatments, but these behaviours occurred less (*p* < 0.05) in tail-docked piglets than those that were castrated. Piglets undergoing the tail-docked treatments and the castration treatment exhibited more behaviours indicative of pain, such as standing longer (*p* < 0.05) with the head lowered in the 60 min after treatment, than those in the sham handling treatment group. There were no treatment effects on cortisol concentrations and behaviour at 23–24 h post-treatment. The physiological results at 30 min post-treatment indicate that tail docking with cauterisation may be less aversive than tail docking with clippers.

## 1. Introduction

Tail biting is potentially a serious welfare problem for the recipient pig. Tail biting involves destructive chewing of the tails of group-mates, with a bleeding bitten tail becoming attractive to other pigs in the group. Tail biting occurs in two stages, a pre-injury and an injury stage, and it is the second stage that results in wounding, bleeding and more severe consequences, such as infection, spinal abscess and paralysis; in extreme cases, death may occur, but tail biting more commonly results in euthanasia or delayed market sales. [1,2]. As a result, the pork producer can incur severe economic losses when the pigs are marketed and, in the meantime, there are obviously serious welfare consequences for the pigs bitten. The etiology of tail biting remains poorly understood and potential factors pre-disposing tail biting are numerous, such as crowding, poor ventilation, breakdown in the food or water supply, poor quality diets and breed type [3,4,5].

Tail docking is a common procedure performed worldwide to reduce tail biting in groups, and there is evidence that the procedure reduces the numbers of tail-bitten pigs [4,5]. In Australia, the Model Code of Practice for the Welfare of Pigs recommends that tail docking be avoided wherever possible. Where tail biting is a problem, all areas of the environment, feeding and management should be investigated to identify contributing factors so that remedial action can be taken, e.g., provision of enrichment to reduce tail biting behaviour [6], and, where tail docking is practiced as a preventative measure, it should be conducted before seven days of age. The EU Council Directive 2008/120/EC prohibits routine tail docking, but the practice is still widely used in many member states. Despite the legal restrictions, in reality, as many as 95% of the pigs in Europe, and approximately 80% of the pigs in UK, are reportedly tail docked [5]. It is estimated that 88% of pigs raised in Australia are tail docked. It is recommended that tail docking should be performed using either side-cutter pliers (clippers) or a cauterising tail docking iron (cauterisation) and that the tail should be docked between 1.5 and 2.5 cm from the base of the tail, in between vertebra [7]. 

There is limited information in the scientific literature on the procedures of tail docking, and whether or not the two common procedures of tail docking differ in terms of both level and duration of pain caused by the procedure [8,9,10,11]. It should be recognised that pain is difficult to study because it is an inherently subjective experience. Behavioural indicators that have been used to assess pain include vocalisation, escape attempts during the procedure, changing postures, such as standing with head lowered, and reduced activity after the procedure [9,12,13,14]. Glucocorticoids are generally accepted as a measure of stress and have also been used to assess pain [8,9,12,15]. Indeed, pain is a powerful stressor, directly stimulating the release of hormones from the hypothalamo-pituitary adrenal axis and sympatho-adrenal medullary system in mammals and birds and their equivalents in fish [16]. However, non-painful components of a surgical husbandry procedure, such as restraint, isolation and the presence of humans, may also increase cortisol concentrations. These traditional behavioural and physiological indices that have been used to study pain are also measures of non-painful stressors. For example, behavioural avoidance and glucocorticoids have been used to assess fear [17,18]. Furthermore, non-painful components of a surgical husbandry procedure, such as restraint, isolation and the presence of humans, may also increase escape behaviour and cortisol concentrations. It should also be recognised that glucocorticoids have anti-inflammatory and immunosuppressive properties in response to tissue injury [17,19]. Nevertheless, while such behavioural and physiological responses are useful measures of the stress response, combining these measures increases the chance of detecting and evaluating the intensity of pain [16]. Neurophysiological responses (activity of the cerebral cortex) of the animal, recorded by electroencephalographic (EEG) responses using a minimal anaesthesia model, have been successfully used to assess nociception in a range of domesticated mammals [20]. 

There is evidence in the scientific literature that surgical castration causes acute pain in piglets [13,14,21]. Piglets that are surgically castrated exhibit a stronger vocal response compared to piglets that are sham castrated or castrated under local anesthesia. Surgical castration also induces activation of the hypothalamic–pituitary–adrenal axis and the sympatho-adrenal medullary system increases vocalisations [13,14,21], and also leads to pain-related behaviours [14]. Therefore, surgical castration was used in this experiment as a negative control and the positive control was a sham handling treatment. A broad examination of the physiological and behavioural responses of piglets was used in this experiment to study the stress responses to the surgical procedures of tail docking and surgical castration. Furthermore, an examination of this stress response and thus the welfare implications, also included behaviour and fitness consequences, such as growth and reproductive efficiency and health [17,18]. This experiment compared the physiological and behavioural responses of two-day-old piglets when they were tail docked using the two common procedures, clippers and cauterisation.

## 2. Materials and Methods

All animal procedures were conducted with prior institutional ethical approval under the requirements of the New South Wales Prevention of Cruelty to Animals Act (1979) in accordance with the National Health and Medical Research Council/Commonwealth Scientific and Industrial Research Organisation/Australian Animal Commission Australian Code of Practice for the Care and Use of Animals for Scientific Purposes (8th edition). 

The experiment was conducted at the Rivalea Australia Research and Innovation Unit, Corowa NSW, Australia. The experiment was conducted between January and March (summer and early autumn). Seventy two sows (Large White × Landrace) and their litters were selected. The sows farrowed in individual farrowing crates. One of four healthy entire male piglets greater than 1.2 kg in live weight was randomly allocated to one of the four following treatments imposed at 2 d post birth (288 entire male piglets in total), and the pigs were individually identified by a number written on their back with a black stock marker. The following treatments were imposed:

1. Sham handling treatment (‘Sham handling’)—piglets were individually held the same way as the other treatments for approximately 30 s, before being returned to their pens.
2. Surgical castration (‘Castration’)—the piglet’s anogenital region was exposed and a scalpel was used to make a 10 mm long incision on each side of the scrotum to expose each testicle, and the testicles were removed by cutting the testicular cord. A disinfectant was applied to the wound and the piglet was immediately returned to its pen.
3. Tail docking using side-cutters (‘Clippers’)—clean, disinfected side-cutters (clippers) were used to cut approximately 2 cm from the base of the tail in between the second and third vertebrae. A disinfectant was applied to the wound and the piglet was returned to its pen.
4. Tail docking using a Stericut^®^ Tail Cauteriser (‘Cauterisation’)—a clean disinfected gas operated Stericut^®^ tail docker was used to cut the tail at the same location as in the clipper treatment. A disinfectant was applied to the wound and the piglet was returned to its pen.

All piglets were individually and gently picked up from their home pen and held, supported under the arm of the technician with their hind area exposed, for approximately 30 s before returning them to their pen. Once the treatments were imposed and the behavioural observations and blood samples were completed at 2 d post-birth, all experimental piglets received a routine iron injection and vaccination.

### 2.1. Cortisol Concentrations

Blood samples were collected by jugular venipuncture. The piglets were picked up in the same order as they were when treatment was imposed. The blood samples were taken at 15 min, 30 min and 24 h post-treatment. The blood sampling was conducted by trained personnel who were able to obtain a blood sample within 20 s of the piglet being picked up. The blood was collected in 2 mL Vacutainer tubes (BD, Franklin Lakes, NJ, USA) treated with lithium heparin, and stored on ice. The individual samples were centrifuged at 7000 rpm and the plasma was poured off and stored frozen at −20 °C until analysed. The samples were assayed for total cortisol at the University of Western Australia. Plasma concentrations of cortisol were measured in duplicate, following the manufacturer’s instructions, by radioimmunoassay, using Immuchem^TM^ Coated Tube Cortisol RIA kits (MP Biomedicals, Belgium). The limit of detection was 0.2 µg/dL. Quality control samples (7.1 and 25.2 µg/dL) were used to estimate inter- (6.4% and 3.8%) and intra-assay (7.4% and 4.8%) coefficients of variation. 

### 2.2. Behaviour

During the imposition of treatment, an escape attempt was defined as a body movement carried out to effect an escape [10]. The duration of vocalisations was also recorded during imposition of treatment.

The behaviours of the four treatment piglets in each litter were videotaped using mounted cameras (Signet Model QV-3063) that enabled viewing the whole farrowing crate. The piglet behaviours observed are described in Table 1 and the duration of these behaviours were sampled as follows. Each of the four piglets per litter was observed continuously for the first 60 s every 5 min in the first 60 min post-treatment. This resulted in four sets of 60-s observations on each of the four piglets in each litter (i.e., a total of 12 min per piglet observed in the first 60 min post-treatment). The duration of these behaviours of the piglets was also measured with this same sampling procedure for 1 h from 23 h post-treatment (i.e., a total of 12 min per piglet 23–24 h post-treatment). 

### 2.3. Tail Lesion Scoring

The tail lesion score was measured on the clipper and cauterisation treatments (as described by Marchant-Forde et al. [11]) at day seven post-treatment and weaning. The lesions were scored from 0 to 5 as follows: 0 = skin intact with no swelling or reddening, complete healing with no scab; 1 = swelling, but skin intact or healing lesion with a scab; 2 = severe swelling, but skin intact or a narrow, red, ulcerated wound around the perimeter of the injury site with little or no exudate, a healing lesion showing a large scab with underlying granulation; 3 = wider band of red, ulcerated skin surrounding injury side, but with no excessive exudate present; 4 = red, ulcerate lesion covered by exudate, swelling of the surrounding tissues; 5 = large, red, ulcerated lesion with much pus and exudate and a strong smell of necrosis, severe swelling.

### 2.4. Growth Performance and the Total Number of Piglets that Died Due to Illness, were Euthanised or were Removed Due To Illness

The piglets were weighed immediately prior to the treatment and then at 7 d post-treatment and at weaning (average of 26 d of age). The total number of piglets that died due to illness, were euthanised or were removed due to illness was recorded. 

### 2.5. Statistical Analysis

Statistical analysis was performed using SPSS (Version 21 -SPSS Inc., Chicago, IL, USA). All data were analysed for normality and data transformation (square root) was performed when required. Behaviours, physiology and growth performance were analysed by using the univariate general linear model, using each piglet as the experimental unit and the sow as the random factor. When significant treatment differences (*p* < 0.05) were detected, least significant difference (LSD) tests were used for pairwise comparisons between all treatments. A Chi-squared analysis was used to analyse the treatments’ effect on the number of piglets that died due to illness, were euthanised or were removed due to illness and placed into recovery pens.

## 3. Results

### 3.1. The Total Number of Piglets that Died Due To Illness, were Euthanised or were Removed Due To Illness

There were no significant treatment effects (X^2^ = 0.70; *p* = 0.951) on the total number of piglets that died due to illness, were euthanised or were removed due to illness. 

### 3.2. Cortisol Concentrations

Cortisol concentrations at 15 min post-treatment were significantly (*p* < 0.05) higher in both tail docking treatments and the surgical castration treatment compared to the sham handling treatment (Table 2). Cortisol concentrations at 30 min post-treatment were significantly (*p* < 0.05) higher in the tail docking with clippers and surgical castration treatments compared to the sham handling treatment, but the cortisol concentrations at 30 min post-treament were more similar in the cauterisation and sham handling treatments. Cortisol concentrations at 15 min and 30 min post-treatment were higher (*p* < 0.05) in the castration treatment than the two tail docking treatments. 

There were no significant treatment effects (*p* > 0.05) on cortisol concentrations at 24 h post-treatment (Table 2).

### 3.3. Behaviour

Scooting and shivering were not observed during the observation period. Playing and frolicking observations were rare and were only observed on five occasions in short bouts. Piglets in the surgical castration treatment vocalised for longer (*p* < 0.05) and performed more escape attempts (*p* < 0.05) than those who received sham handling or tail docking treatments. Piglets undergoing the tail docking treatments exhibited more vocalisations (*p* < 0.05) and performed more escape attempts during treatment (*p* < 0.05) than those undergoing the sham handling treatment (Table 3). Piglets in both tail docking treatments and the castration treatment spent more time standing with their head lowered compared to the sham handling treatment piglets (*p* < 0.05). There was a trend (*p* = 0.055) for the piglets in the castration treatment group to spend less time lying in contact with the sow compared to those in the other treatments.

There were no significant treatment effects (*p* > 0.05) on the behaviours observed 23–24 h post-treatment (Table 4).

### 3.4. Tail Lesion Scoring

There were no significant treatment effects (*p* > 0.05) on the tail lesion scores of the piglets at 7 d post-treatment (1.3 in both clipper and cauterisation treatments) and at weaning (0.03 and 0.0 in the clipper and cauterisation treatments, respectively).

### 3.5. Growth Performance

There were no significant treatment effects (*p* > 0.05) on the live weights of the piglets prior to treatment or at 24 h post-treatment, 7 d post-treatment or weaning. There were no significant treatment effects (*p* > 0.05) in the rate of gain of piglets from 0 to 24 h post-treatment, 0 to 7 d post-treatment or treatment to weaning (Table 5).

## 4. Discussion

This experiment used a broad examination of physiological and behavioural responses of piglets to examine their stress responses to tail docking and surgical castration. The results show that in comparison to the sham handling treatment, surgical castration of two-day-old piglets caused greater acute behavioural and physiological responses. Surgical castration increased the duration of vocalisations, escape attempts during treatment and cortisol concentrations at 15 and 30 min after treatment. Surgically castrated pigs also spent more time with their heads lowered and idle during the first 60 min after treatment. These results are consistent with those found in other studies investigating surgical castration [12,17,23,24,25]. Interestingly, in the present experiment, these behavioural and physiological differences were not apparent after 23–24 h. In contrast, other studies have shown that changes in behaviours, such as reduced time massaging the udder, time inactive, tail wagging and scratching the rump, indicate that piglets may experience pain for up to 4 d after surgical castration [12,23].

Tail docking using either clippers or a cauteriser in the present experiment increased the piglets’ cortisol concentrations at 15 and 30 min post-treatment compared to the sham handling treatment. However, the cortisol concentrations at 30 min post-treatment were lower after the cauterisation treatment than the clipper treatment which indicates that cauterisation may be less aversive than the clipper treatment. Sutherland et al. [8] found that piglets tail docked with clippers had higher cortisol concentrations after 60 min than piglets that were tail docked with cauterisation or handled only. The cortisol concentrations of piglets in all three treatments were similar at 90 min post-treatment in this previous experiment. Care needs to be taken in comparing studies as the pigs in the experiment by Sutherland were older (6 d of age) and the male pigs had been surgically castrated approximately 3 d prior. Prunier et al. [7] also showed that cortisol concentrations did not differ between handled piglets and those that had been tail docked by cauterisation for up to 180 min after tail docking. The findings of these three experiments are in general similar. Furthermore, the cortisol concentrations in the piglets in the four treatments in the current experiment were similar at 24 h post-treatment, indicating that the cortisol response had diminished.

Piglets in the two tail docking treatments exhibited increases in behaviours indicative of pain both during the treatment imposition and in the first 60 min after treatment. Tail-docked piglets vocalised for longer and displayed more escape responses during treatment than piglets in the sham handling treatment. However, the duration of vocalisations and number of escape attempts were lower in tail-docked piglets than those surgically castrated. Furthermore, piglets in the two tail docking treatments spent more time standing with their heads lowered in the first 60 min post-treatment than those in the sham handling treatment which is indicative of increased stress after tail docking and castration than after the sham handling treatment. Although cauterisation induced less of a cortisol response 30 min after treatment compared to clipping, the behavioural responses in the first 60 min post-treatment were similar in the two treatments. The finding that these behavioural responses in the tail docking and castration treatments at 23–24 h post-treatment were similar to those of the sham handling treatment suggests that pain had diminished by this time. 

There was no difference in the tail lesion scores between the clipper and cauterisation treatments at 7 d post-treatment and weaning. The average lesion score at d 7 indicated some swelling but intact skin and healing lesions with a scab, and by weaning, there was intact skin with no swelling or reddening and a complete healing with no scab. These results suggest that there is no difference between the two tail docking procedures in terms of wound healing after docking and that both procedures result in acceptable wound healing. The long-term detrimental implication of cauterisation, such as the formation of neuromas when the nociceptors regenerate (as found by Simonsen et al. [7]) requires further investigation. Herskin et al. [26] showed that tail docking by cauterisation caused the formation of neuromas on the tail stump. Eicher et al. [27] showed that the tail stumps of heifers that had been docked with a cauteriser were more sensitive to heat and cold. As part of the present long-term project, a recent study assessing tail nerve histomorphology post-slaughter of pig tails docked using a clipper or cauterisation procedure indicated that tail docking using either procedure results in neuroma formation, and that both procedures have the potential to affect long-term pig welfare [28].

There were no adverse effects of the two tail docking procedures and surgical castration on growth performance in the present experiment. It is well known that activation of the hypothalamic–pituitary–adrenal axis, particularly in the long-term, can lead to suppression of growth hormones and glucocorticosteroids can induce resistance to growth factors in target tissues [29]. Glucocorticosteroids and adrenocorticotrophic hormones can also have a catabolic effect on the body [30]. Although there was evidence of an acute cortisol response in the castrated and tail-docked piglets, the duration and magnitude of the stress response appeared to be insufficient to affect the efficiency of growth. There were also no adverse effects of the two tail docking procedures and surgical castration on health on the basis of the total number of piglets that died due to illness, were euthanised or were removed due to illness.

## 5. Conclusions

In conclusion, tail docking two-day-old piglets using clippers or a cauterisation procedure caused an increased cortisol response at 15 and 30 min post-treatment in comparison to handling alone. Tail docking also caused an increase in the duration of vocalisations, the number of escape attempts during treatment and the duration of standing with head lowered in the first 60 min after treatment. However, the impact on stress physiology and behaviour of these procedures diminished by 23–24 h post-treatment. Furthermore, based on cortisol concentrations, cauterisation appeared to be less aversive than the clipper procedure. 

## Figures and Tables

**Table 1:** Descriptions of the piglet behaviours observed (modified from Hay et al. and Hurnick et al. [12,22]).

Behaviours	Description
Standing	Upright position with bodyweight supported by all four legs.
Standing with head lowered	Upright position with bodyweight supported by all four legs. Head lower than shoulders.
Sitting	Body weight supported by the hind-quarters and front legs.
Lying (with sow contact)	Maintaining a recumbent position in contact with a part of the sow.
Lying (without sow contact)	Maintaining a recumbent position not in contact with a part of the sow.
Idle	Not performing any behaviour.
Walking	Relatively low speed locomotion in which propulsive force derives from action of legs.
Massaging udder/Nursing	Nose in contact with the udder/teat in mouth. Vigorous and rhythmic suckling movements.
Asleep	Eyes closed while lying down.
Playing/frolicking	Head shaking, springing (sudden jump or leap), running with horizontal and vertical bounces.
Scooting	Causal part of body being dragged across ground.
Scratching	Scratching the rump against the floor or walls of the pen.
Shivering	Shivering as with cold.

**Table 2:** Effect of treatment on cortisol concentrations at 15 and 30 min and 24 h post-treatment.

Measurement	Sham Handling	Castration	Clippers	Cauterisation	SEM *	*p* Value
Cortisol (ng/mL)						
15 min	91.2 ^a^	128.9 ^c^	110.7 ^b^	106.4 ^b^	2.20	0.000
30 min	115.3 ^a^	145.8 ^c^	126.0 ^b^	121.8 ^ab^	1.86	0.000
24 h	49.0	45.3	42.0	44.0	1.92	0.536

* Standard Error of Mean; ^abc^ Within-row values with different superscripts are significantly different (*p <* 0.05; Fisher’s least significant difference (LSD) test).

**Table 3:** Effect of treatments on duration of vocalisations and number of escape attempts during imposition of treatments and durations of behaviours of piglets for the first 60 min after treatment.

Variable **	Sham Handling	Castration	Clippers	Cauterisation	SEM *	*p* Value
*At treatment imposition* Duration of vocalisations during treatment (s)	1.6 ^a^ (2.6)	3.9 ^c^ (15.2)	2.0 ^b^ (4.0)	2.0 ^b^ (4.0)	0.07	0.000
Number of escape attempts during treatment	1.7 ^a^ (2.9)	4.1 ^c^ (16.8)	2.0 ^b^ (4.0)	2.1 ^b^ (4.4)	0.07	0.000
*First 60 min after treatment* Standing (normal) (s)	17.3 (299.3)	17.0 (289.0)	16.4 (269.0)	16.5 (272.3)	0.22	0.604
Standing (head lowered) (s)	2.3 ^a^ (5.3)	4.8 ^b^ (23.0)	4.3 ^b^ (18.5)	4.1 ^b^ (16.8)	0.26	0.007
Sitting (s)	1.5 (2.3)	2.1 (4.4)	1.5 (2.3)	1.9 (3.6)	0.18	0.565
Lying (with sow contact) (s)	9.2 (84.6)	7.1 (50.4)	9.1 (82.8)	8.3 (68.9)	0.41	0.055
Lying (without sow contact)	14.1 (198.8)	15.1 (228.0)	13.5 (182.3)	14.7 (216.1)	0.38	0.436
Idle (s)	10.7 (114.5)	12.8 (163.8)	11.4 (130.0)	11.0 (121.0)	0.24	0.160
Walking (s)	9.6 (92.2)	8.7 (75.7)	8.8 (77.4)	9.3 (86.5)	0.17	0.199
Massaging udder/Nursing (s)	12.3 (151.3)	11.3 (127.7)	12.1 (146.4)	12.4 (153.8)	0.33	0.749
Asleep (s)	16.8 (282.2)	16.6 (275.6)	16.4 (269.0	16.2 (262.4)	0.24	0.902

^abc^ Within-row values with different superscripts are significantly different (*p* < 0.05). * Standard Error of Mean. ** All variables were square root transformed prior to statistical analysis. Transformed means are presented, and back transformed means are presented in parentheses.

**Table 4:** Effect of treatment on behaviour of piglets for 23–24 h after treatment **.

	Sham Handling	Castration	Clippers	Cauterisation	SEM *	*p* Value
Standing (normal) (s)	10.3 (106.1)	10.3 (106.1)	10.3 (106.1)	10.4 (108.2)	0.29	0.996
Standing (head lowered) (s)	0.7 (0.5)	0.9 (0.8)	0.8 (0.64)	1.3 (1.7)	0.14	0.448
Sitting (s)	1.4 (2.0)	1.2 (1.4)	1.2 (1.4)	0.5 (0.25)	0.16	0.397
Lying (with sow contact) (s)	10.3 (107.0)	8.2 (67.2)	9.4 (88.4)	8.9 (79.2)	0.56	0.622
Lying (without sow contact) (s)	17.3 (299.3)	19.4 (376.4)	18.8 (353.4)	19.0 (361.0)	0.48	0.393
Idle (s)	5.1 (26.0)	5.6 (31.3)	6.0 (36.0)	4.9 (24.0)	0.27	0.410
Walking (s)	3.9 (15.2)	4.3 (18.5)	4.1 (16.8)	4.2 (17.6)	0.23	0.962
Massaging udder/Nursing (s)	10.7 (114.5)	10.1 (102.0)	10.8 (116.6)	10.4 (108.2)	0.30	0.787
Asleep (s)	22.0 (484.0)	22.1 (488.4)	22.1 (488.4)	22.4 (501.8)	0.19	0.868

* Standard Error of Mean. ** All data were square root transformed prior to statistical analysis. Transformed means are presented, and back transformed means are presented in parentheses.

**Table 5:** Effect of treatment on growth performance of piglets.

	Sham Handling	Castration	Clippers	Cauterisation	SEM *	*p* Value
Live weight pre- treatment (kg)	1.9	2.0	1.9	1.9	0.02	0.281
Weaning weight (kg)	8.0	7.7	7.3	7.7	0.13	0.273
Growth rate (g/d) 0–24 h post-treatment	151.1	128.9	135.4	127.4	6.94	0.611
Growth rate (g/day) 0–7 d post-treatment	228.9	216.1	200.7	215.1	4.97	0.259
Growth rate treatment-weaning (g/d)	223.0	207.6	199.7	207.9	4.09	0.235

* Standard Error of Mean.
